# Genetic predisposition of the gastrointestinal microbiome and primary biliary cholangitis: a bi-directional, two-sample Mendelian randomization analysis

**DOI:** 10.3389/fendo.2023.1225742

**Published:** 2023-10-12

**Authors:** Xin Luo, Xin You

**Affiliations:** ^1^Department of Rheumatology and Clinical Immunology, Peking Union Medical College Hospital, Chinese Academy of Medical Sciences & Peking Union Medical College, Beijing, China; ^2^Key Laboratory of Rheumatology & Clinical Immunology, Ministry of Education, Beijing, China; ^3^National Clinical Research Center for Dermatologic and Immunologic Diseases (NCRC-DID), Beijing, China

**Keywords:** gastrointestinal microbiome, primary biliary cholangitis, Mendelian randomization, genetic predisposition, autoimmune liver disease

## Abstract

**Background:**

The gut-liver axis indicates a close relationship between the gastrointestinal microbiome (GM) and primary biliary cholangitis (PBC). However, the causality of this relationship remains unknown. This study investigates the causal relationship between the GM and PBC using a bidirectional, two-sample Mendelian randomization (MR) analysis.

**Methods:**

Genome-wide association data for GM and PBC were obtained from public databases. The inverse-variance weighted method was the primary method used for MR analysis. Sensitivity analyses were conducted to assess the stability of the MR results. A reverse MR analysis was performed to investigate the possibility of reverse causality.

**Results:**

Three bacterial taxa were found to be causally related to PBC. Class Coriobacteriia (odds ratio (OR) = 2.18, 95% confidence interval (CI): 1.295-3.661, P< 0.05) and order Coriobacteriales (OR = 2.18, 95% CI: 1.295-3.661, P<0.05) were associated with a higher risk of PBC. Class Deltaproteobacteria (OR = 0.52, 95% CI: 0.362–0.742, P< 0.05) had a protective effect on PBC. There was no evidence of reverse causality between PBC and the identified bacterial taxa.

**Conclusion:**

Previously unrecognized taxa that may be involved in the pathogenesis of PBC were identified in this study, confirming the causality between the GM and PBC. These results provide novel microbial targets for the prevention and treatment of PBC.

## 1 Introduction

Primary biliary cholangitis (PBC) is a type of autoimmune liver disease with the clinical features of a high titer of anti-mitochondrial antibody (AMA) in the serum, elevated biliary enzymes, and specific bile duct pathology (1). Anatomically, there is bidirectional crosstalk between the intestine and liver, suggesting that various intestinal-derived products (such as nutrients, bile acids, and bacterial metabolites) are transported to the liver through the portal vein and that the liver secretes bile and antibodies into the intestine. This bidirectional relationship is termed the gut-liver axis, which is the theoretical basis for the relationships between diseases and the gastrointestinal microbiome (GM) (2).

Gastrointestinal barrier dysfunction and alterations in the gut microbial composition are commonly observed in patients with chronic liver diseases. Substances produced by the GM, such as bacterial components and microbial metabolites, accumulate in the liver through the gut-liver axis (3, 4). Specifically, small molecular motifs derived from the GM, such as lipopolysaccharide, are detected by innate immune receptors such as Toll-like receptors. This recognition triggers innate immune responses that ultimately lead to changes in the liver immune microenvironment, resulting in chronic inflammation and, in some cases, progression to hepatocellular carcinoma (5, 6). Microbial metabolites, including secondary bile acids, short-chain fatty acids, and ethanol, are produced through fermentation of intestinal contents by the GM (7). These metabolites have various effects on the liver, including direct toxic effects, pro-inflammatory or anti-inflammatory immune responses, and the maintenance of intestinal epithelial cell stability via the gut-liver axis (8–11).

Changes to the GM are observed in patients with PBC. Compared with healthy controls, the fecal microbial α-diversity is decreased in patients with PBC (12, 13). Several studies have reported depletion of potential probiotics and enrichment of opportunistic pathogenic bacteria within the GM of patients with PBC (12, 14, 15). Disturbance of the GM is a pivotal factor in the progression of PBC. However, previous studies report both consistent and conflicting results as they are case-control observational studies. Although these studies controlled for the effects of age and sex as much as possible, the GM is susceptible to environmental, dietary pattern, and lifestyle changes. The influence of confounding factors (16) renders these factors difficult to control. In addition, due to the bidirectional relationship of the gut-liver axis, it is unclear whether an altered GM triggers PBC or is a reflection of disease status (17).

Mendelian randomization (MR) is a new technique used to explore causal associations using genetics. Genetic variants are used to construct instrumental variables (IVs) representing exposures, which are then used to estimate the causal association between exposures and an outcome (18). Due to the random allocation of genotypes from parents to offspring, the association between genetic variants and outcomes remains unaffected by common confounding factors, establishing reasonable causality (19).

In this study, two-sample MR analyses were conducted to assess the potential causal relationships between bacterial taxa (used as exposures) and PBC (used as the outcome) using genome-wide association study (GWAS) summary statistics from the MiBioGen and GWAS Catalog public databases. The reverse causal relationships between each identified bacterial taxon and PBC were also investigated.

## 2 Methods

### 2.1 Study design and data sources

**Figure 1:**
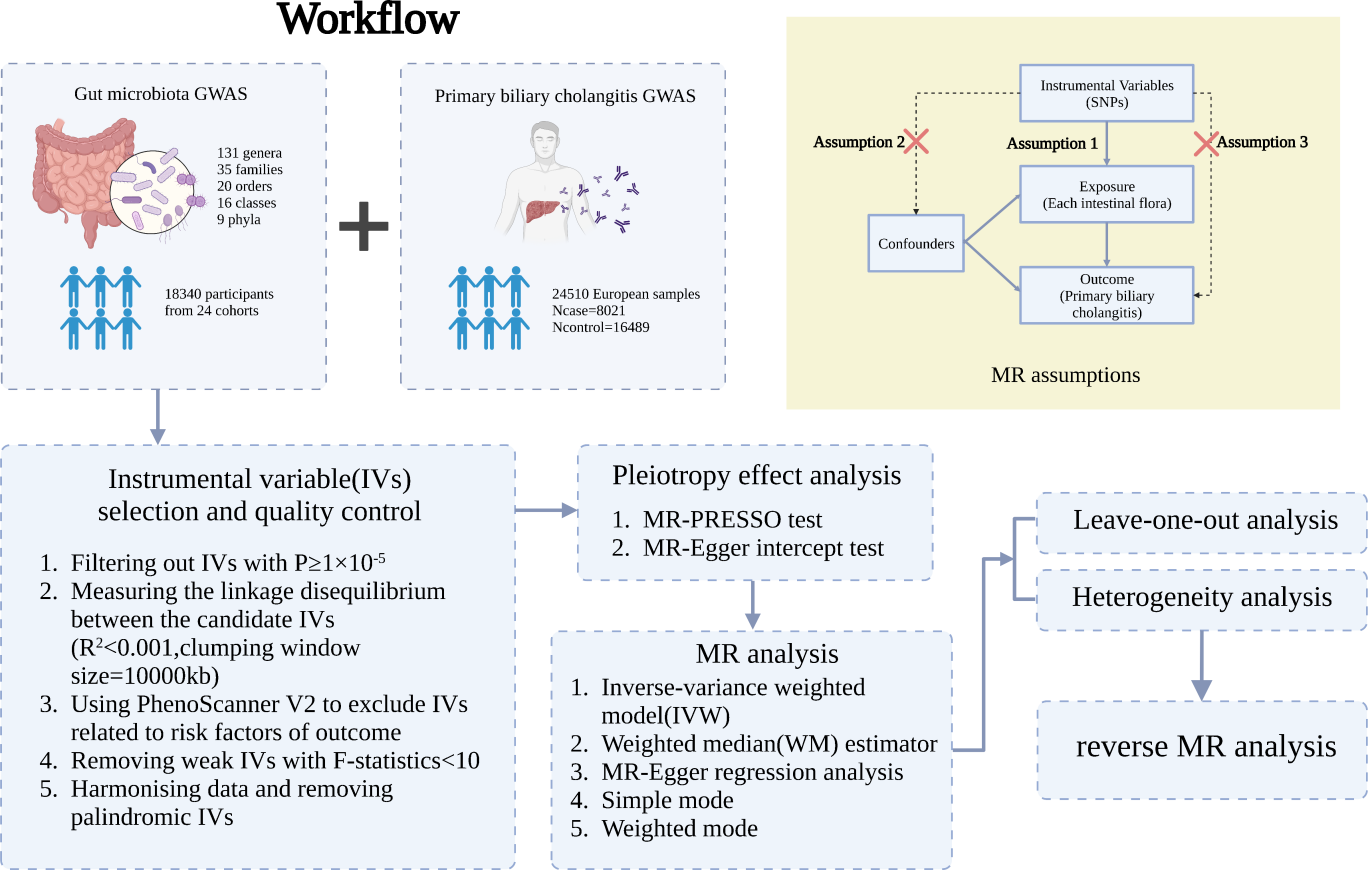
Three assumptions of two-sample Mendelian randomization (MR) analysis and the workflow of this study. Concisely, each bacterial taxon (at phylum, class, order, family, and genus) served as an exposure, and PBC served as the outcome. Quality control was taken to ensure that the selected IVs were reliable and accurate in determining a causal link between GM and PBC.

A two-sample MR analysis satisfies three assumptions (20): the IVs chosen from the datasets are related to the exposure; there is no association between the IVs and confounders of the exposure-outcome relationship; and the IVs are linked to the outcome through exposures rather than any other way (**Figure 1**). Single-nucleotide polymorphisms (SNPs) of each bacterial taxon were screened using the MiBioGen database to be used as IVs (21, 22). According to the previous publication (22), once the quality control-filtered merged reads are processed, all cohorts can use the standardized 16S processing pipeline available at the GitHub repository (23). The cohorts used in this study are detailed in the Supplementary Materials of the previous publication (22). The overall proportion of proton pump inhibitor and antibiotic usage was less than 10% in this study. Most of the fecal samples were gender- and age-matched, and sample quality control, such as removing ethnic outliers and sex mismatches, was performed prior to the GWAS analysis. Outcome data were obtained from the latest genome-wide meta-analysis of PBC (24). Each data source included in the analyses is detailed in **Table 1**.

**Table 1:** Description of the data sources.

Data	GWAS^a^ summary data of each bacterial taxon	GWAS summary data of PBC^b^
Data source	MiBioGen (21, 22)	GWAS Catalog (24)(ID: GCST90061440)
Setting	Meta-analysis study Population: European, Asian, and North American (mainly European)	Meta-analysis study Population: European
Participants	24 cohorts from 18340 participants, containing 211 taxa (131 genera, 35 families, 20 orders, 16 classes, and nine phyla)	24510 European (Canada, U.S., Italy, U.K.) Number of PBC = 8021 Number of controls = 16,489
Measurement, quality control, and selection of SNPs^c^ (when used as exposures)	Minor allele frequency > 0.05 P< 1×10^-5^ Measurement of linkage disequilibrium (R2<0.001, clumping window size = 10000kb) Excluding SNPs related to risk factors of outcome F-statistics ≥10 Removing palindromic and ambiguous SNPs	Minor allele frequency > 0.1 P< 5×10^-8^ Measurement of linkage disequilibrium (R2<0.001, clumping window size = 10000kb) Excluding SNPs related to risk factors of outcome F-statistics ≥10 Removing palindromic and ambiguous SNPs
Methods of assessment or diagnostic criteria for diseases	The core-measurable microbiome is defined as the list of bacterial taxa present in more than 10% of the samples in a cohort.	All patients fulfilled the criteria of the European Association for the Study of Liver Diseases for primary biliary cirrhosis.

^a^, genome-wide association; ^b^, primary biliary cholangitis; ^c^, single nucleotide polymorphisms.

### 2.2 Instrumental variable selection and quality control

SNPs with a less stringent cutoff of P < 1×10^−5^ were considered significantly related to GM and chosen as the candidate IVs. This strategy increased the number of SNPs available for subsequent analyses (25–27). The linkage disequilibrium (LD) between the candidate IVs were calculated using the 1000 Genomes Project European sample data as the reference panel (LD correlation coefficient set to R^2^< 0.001 and clumping window size = 10,000 kb). Then, the PhenoScanner V2 (28, 29) was used to search the candidate IVs for confounders to avoid horizontal pleiotropy. IVs that correlated with risk factors for PBC were excluded. The F-statistic was used to identify any IV bias, and IVs with an F-statistic< 10 were eliminated (30, 31) by applying formula used in a previous study (**Appendices Equations A, B**) (31, 32). Last, the exposure and outcome of the SNPs were harmonized to confirm that the effect alleles of the SNPs on the exposure matched with the identical effect alleles on the outcome. Unmatched SNPs were removed from the analyses. Palindromic and ambiguous SNPs were inferred during the harmonization process and also removed.

### 2.3 Pleiotropy effect analysis

MR-PRESSO (NbDistribution = 10000) and MR-Egger regression tests were used to detect potential horizontal pleiotropy effects. The MR-PRESSO outlier test assessed the pleiotropic significance of individual SNPs, whereas the MR-PRESSO global test provided an overall p value for horizontal pleiotropy. The SNPs were sorted based on their MR-PRESSO outlier test p values in ascending order and eliminated individually. Each time an SNP was removed, the MR-PRESSO global test was performed for the remaining SNPs. This procedure was repeated until the global test p value was no longer statistically significant (P > 0.05). All pleiotropic SNPs were removed prior to the MR analysis.

### 2.4 MR analysis

MR results based on fewer than three shared SNPs were excluded. The common methods used for causal inference were the inverse-variance weighted (IVW) method (the main approach for causal detection in two-sample MR analysis without horizontal pleiotropy (33)), weighted median (WM) method (34), MR-Egger regression method (20), simple mode (35), and weighted mode (36). The IVW method is complimented by the other methods, which expands the range of confidence intervals (37). A previously-reported multiple testing significance threshold at each feature level (phylum, class, order, family, and genus), defined as P< 0.05/n (where n is the effective number of independent bacterial taxa at the corresponding taxonomic level), was used (38). Sensitivity analyses were performed to assess the robustness of the findings. A leave-one-out analysis was conducted to determine if any single SNP drove the significant results (39).

### 2.5 Heterogeneity

The heterogeneity of the IVs was measured using Cochran’s IVW Q statistics. A Q value higher than the number of instruments minus one suggests the presence of invalid instruments and heterogeneity. A Q statistic p value< 0.05 indicates heterogeneity (40, 41).

### 2.6 Reverse MR analysis

A reverse MR analysis was conducted to investigate the causal influence of PBC on each identified bacterial taxon that was identified as significant in the previous analyses. PBC was used as the exposure (the p value for SNPs significantly related to PBC was set to 5×10^–8^), and each identified causal bacterial taxon was set as the outcome. The reverse MR analysis was conducted using the same steps as the quality control of the IVs, MR analysis, and sensitivity analysis.

All statistical analyses were performed using R software (42) (version 4.1.3). The R packages used for the statistical analysis were TwoSampleMR (35, 39) (version 0.5.6) and MR-PRESSO (43) (version 1.0).

## 3 Results

### 3.1 Selection of IVs

A total of 14570 SNPs among 211 taxa were identified, including 937, 1583,1642, 2567, and 7841 at the phylum, class, order, family, and genus levels, respectively (**Table S1**). After clumping, 2212 SNPs remained as candidate IVs. The PhenoScanner V2 identified only one candidate IV (**Table S2**) that was associated with smoking, a risk factor for PBC (1). The F-statistics of the IVs were all > 10 (**Table S3**), suggesting no indication of weak instrument bias. A total of 711 IVs remained after harmonizing the exposure and outcome data (**Table S4**). Three pleiotropic SNPs were removed via the MR-PRESSO outlier test (**Table S5**).

### 3.2 Causal associations between the GM and PBC

The MR results were retained if they were based on three or more shared SNPs. A total of 149 independent bacterial taxa remained after the MR analysis, including 88 at the genus level (P threshold of 5.68×10^−4^), 27 at the family level (P threshold of 1.85×10^-3^), 14 at the order level (P threshold of 3.57×10^-3^), 12 at the class level (P threshold of 4.17×10^-3^), and eight at the phylum level (P threshold of 6.25×10^-3^) (**Table S6**).

**Figure 2:**
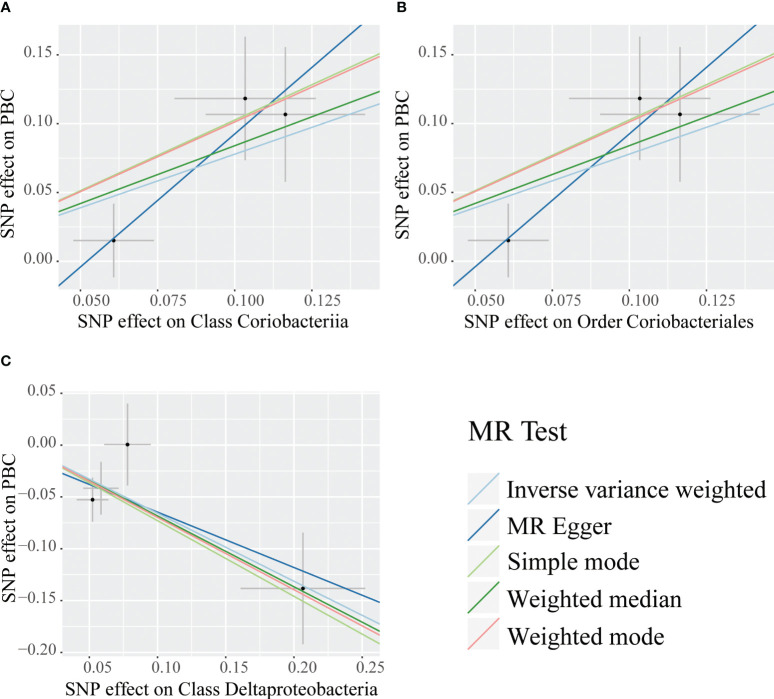
Scatter plots for the causal association between bacterial taxa and primary biliary cholangitis. Single nucleotide polymorphisms (SNPs) were used to assess the impact of each bacterial taxon on primary biliary cholangitis (PBC) by five MR methods **(A–C)**. The dots represent the effect size (β) of each SNP on each bacterial taxon (x-axis) and PBC (y-axis), and the grey crosses represent the standard errors. Regression slopes show the estimated causal effect of each bacterial taxon on PBC. The light blue, dark blue, light green, dark green, and red regression lines represent the inverse variance weighted method, MR-Egger regression, simple mode, weighted median method, and weighted mode, respectively.

**Figure 3:**
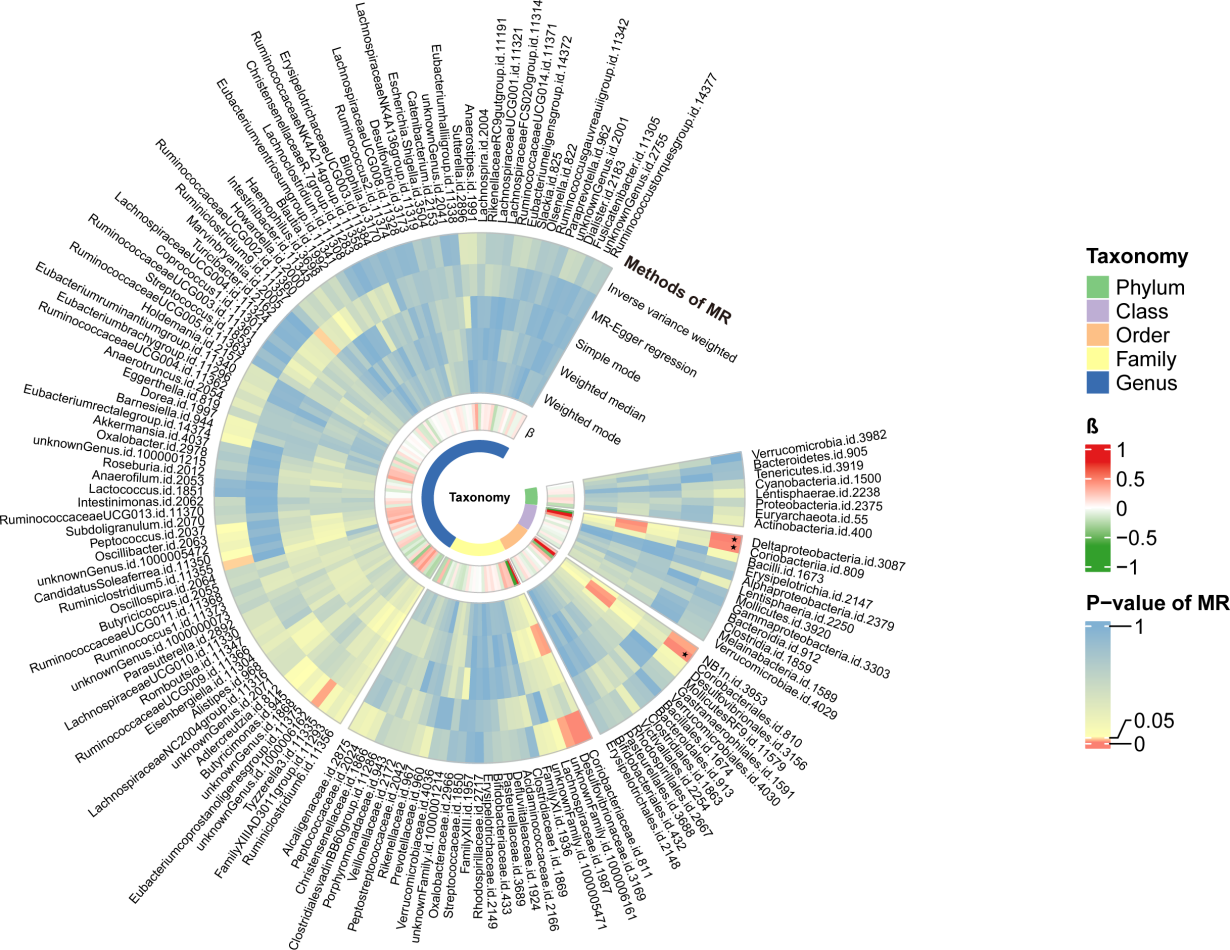
The circular heat map of two-sample Mendelian randomization (MR) analysis. The first track represented the p-value of the MR analysis. From external to internal, it represented different methods, including the inverse variance weighted method, MR-Egger regression, Simple mode, Weighted median, and weighted mode method. The color gradient tended towards red to indicate smaller p-values. The block marked with ‘black stars’ signified that the taxon reached the modified significance threshold at a specific taxonomy. The second track represented the β value. Red blocks indicated harmful factors for PBC, with darker shades indicating a higher risk of illness. Green blocks represented protective factors against PBC, with darker shades indicating a lower risk of illness.

Causal relationships between PBC and three bacterial taxa, class Coriobacteriia, order Coriobacteriales, and class Deltaproteobacteria, were identified using the IVW and WM methods (P< 0.05) (**Table 2**, **Figures 2**, **3**). The p values of the IVW method were inferior to the corresponding modified thresholds. The results of the IVW method indicate that the class Coriobacteriia (odds ratio (OR) = 2.18, 95% confidence interval (CI): 1.295-3.661, P = 3.33×10^-3^) and order Coriobacteriales (OR = 2.18, 95% CI: 1.295-3.661, P = 3.33×10^-3^) are associated with a higher risk of PBC and that the class Deltaproteobacteria (OR = 0.52, 95% CI: 0.362–0.742, P = 3.25×10^−4^) has a protective effect on PBC. The MR-Egger regression, simple mode, and weighted mode methods yielded similar causal estimates for the magnitude and direction.

**Table 2:** The causal effects of bacterial taxa on primary biliary cholangitis.

Taxa	Method	N^a^	β^b^	SE^c^	p-value	OR^d^	95% CI^e^
Class Coriobacteriia	MR Egger	3	1.93	8.64×10^-1^	2.68×10^-1^	6.91	1.27-37.536
	Weighted median	3	8.40×10^-1^	3.32×10^-1^	1.14×10^-2^	2.32	1.208-4.44
	Inverse variance weighted	3	7.78×10^-1^	2.65×10^-1^	3.33×10^-3^	2.18	1.295-3.661
	Simple mode	3	1.03	4.57×10^-1^	1.54×10^-1^	2.79	1.14-6.829
	Weighted mode	3	1.01	4.69×10^-1^	1.63×10^-1^	2.75	1.099-6.894
Order Coriobacteriales	MR Egger	3	1.93	8.64×10^-1^	2.68×10^-1^	6.91	1.27-37.536
	Weighted median	3	8.40×10^-1^	3.32×10^-1^	1.15×10^-2^	2.32	1.207-4.442
	Inverse variance weighted	3	7.78×10^-1^	2.65×10^-1^	3.33×10^-3^	2.18	1.295-3.661
	Simple mode	3	1.03	4.08×10^-1^	1.28×10^-1^	2.79	1.254-6.21
	Weighted mode	3	1.01	4.26×10^-1^	1.41×10^-1^	2.75	1.193-6.347
Class Deltaproteobacteria	MR Egger	4	-5.35×10^-1^	3.97×10^-1^	3.11×10^-1^	0.59	0.269-1.277
	Weighted median	4	-6.83×10^-1^	2.46×10^-1^	5.43×10^-3^	0.50	0.312-0.817
	Inverse variance weighted	4	-6.57×10^-1^	1.83×10^-1^	3.25×10^-4^	0.52	0.362-0.742
	Simple mode	4	-7.29×10^-1^	3.38×10^-1^	1.20×10^-1^	0.48	0.249-0.935
	Weighted mode	4	-6.98×10^-1^	2.83×10^-1^	9.04×10^-2^	0.50	0.286-0.867

^a^ Numbers of SNPs, ^b^ Coefficient in the regression model, ^c^ Standard error, ^d^ Odds ratio, ^e^ Confidence interval.

### 3.3 Sensitivity analysis

**Figure 4:**
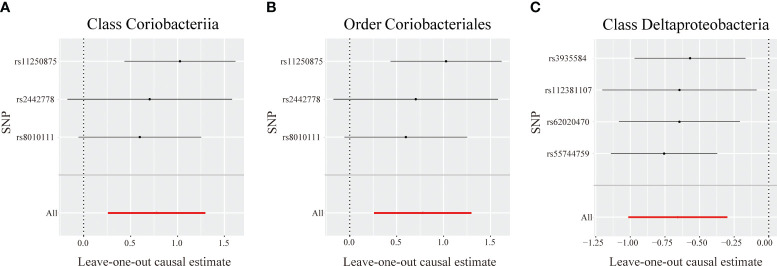
Leave-one-out analysis for identified bacterial taxa on primary biliary cholangitis. The sensitivity of the causal effect of different single nucleotide polymorphisms (SNPs) of each taxon on primary biliary cholangitis was analyzed through leave-one-out analysis in **(A–C)**. The error bar depicts the 95% confidence interval using the inverse variance weighted method.

The MR-Egger intercept indicated no horizontal pleiotropy in the identified taxa (P > 0.05). The MR-PRESSO analysis revealed no outliers among the IVs. Additionally, the Cochrane Q statistics indicated no noticeable heterogeneity (P > 0.05) (**Table S7**). The leave-one-out analysis demonstrated that no individual SNP significantly affected the correlation between each identified bacterial taxon and PBC (**Figure 4**).

### 3.4 Reverse MR analysis

No reverse causal association between PBC and the identified bacteria were identified (**Table S8**).

## 4 Discussion

Researchers have reported that the GM contributes to PBC via molecular mimicry, translocation of gut bacteria to the liver through the damaged intestinal epithelium, movement of immune cells from the intestine to the liver, and abnormal bile acid metabolism (44, 45). Molecular mimicry is a common mechanism by which foreign substances, such as the GM, cause autoimmunity in the body. This occurs when proteins or peptides from the gut microbiota resemble self-peptides, which may activate T or B cells that attack host cells in vulnerable individuals (46). AMA is an autoantibody specific for PBC that targets lipoic acid on 2-oxo-acid dehydrogenase complexes within the inner mitochondrial membrane. Increases in the number of autoreactive clusters of CD4^+^or CD8^+^ pyruvate dehydrogenase complex (PDC-E2)-specific T cells are observed in the liver (1). There is growing evidence that microbial mimics can induce AMA expression. Bacterial sequences from *Escherichia coli* and *Sphingomonas* can react with AMA in the serum of patients with PBC (47, 48), and their ability to induce autoimmune cholangitis has been verified *in vivo* (49, 50). No causal relationship between *Escherichia coli* or *Sphingomonas* and PBC was identified in this study, which may be due to the limited number of taxa in the MiBioGen database; therefore, the corresponding IVs could not be screened for the MR analyses.

In this study, the class Coriobacteriia and its lower taxonomic rank order Coriobacteriales of the phylum Actinobacteria (51) were associated with a higher risk of developing PBC. Members of this category are anaerobic organisms that survive in various ecological environments and do not produce spores. They may either be strict or facultative in their anaerobic requirements (52–55). Limited research regarding their influence on human diseases and the relationships between the class Coriobacteriia, order Coriobacteriales, and PBC has been conducted. Yi et al. reported that long-term exposure to nitrogen dioxide significantly increases gamma-glutamyl transpeptidase and glutamic-pyruvic transaminase levels in patients with schizophrenia and that Coriobacteriales intestinal bacteria mediates this effect by 13.98% and 49.56%, respectively (56). The results of this previous study suggest that Coriobacteriales may be an intermediary in the mechanism by which smoking or environmental exposure initiates PBC (1). Another study reported that Coriobacteriaceae and Coriobacteriales are significantly enriched in the urethral secretions of patients with chronic prostatitis (57), a specific type of urinary tract infection. In addition, several extensive case-control cohort studies have reported that urinary tract infections are related to PBC (1), which is supported by the genetic results of the current study. Approximately 75-95% of patients with PBC suffer from hyperlipidemia due to various complex procedures associated with biliary cholestasis (58, 59). Coriobacteriia were elevated in the guts of females with low high-density lipoprotein cholesterol (60). Interestingly, genera in the Coriobacteriia class were lower in patients with familial hypercholesterolemia who had used statins for more than 12 months than in healthy control patients (61). Patients with PBC and metabolic syndrome are at a higher risk of cardiovascular events (62). However, further experimental verification is required to determine whether Coriobacteria can be targeted to improve the lipid metabolism abnormalities in patients with PBC.

In this study, the class Deltaproteobacteria was found to have a defensive role in preventing PBC. Deltaproteobacteria is a gram-negative class of Proteobacteria involved in the carbon and sulfur cycles (63). The lower taxonomic levels of the class Deltaproteobacteria, order Desulfovibrionales, and family Desulfovibrionaceae tended to have a protective effect on PBC, though the threshold of the modified p-value was not met in this study (**Figure 3**, **Table S6**). In contrast to the current findings, previous studies suggested that Deltaproteobacteria and its descendants are potentially pathogenic gut bacteria (64–70). Desulfovibrionaceae is capable of producing hydrogen sulfide (H_2_S) (71). Excessive H_2_S in the focal intestinal tract may reduce disulfide bonds in the mucous layer, breaking down the mucous barrier and exposing epithelial cells to bacteria and toxins, which may lead to intestinal inflammation (72). In contrast, low levels of endogenous or exogenous H_2_S directly stabilize the mucus layers, preventing microbial biofilms from attaching to the epithelium, which stops the release of harmful pathogenic microorganisms and assists in resolving tissue damage and inflammation (72). In addition, metabolic H_2_S has been reported to improve insulin resistance in mice with non-alcoholic fatty liver disease via the AKT signaling pathway (73), suggesting that an overabundance of Deltaproteobacteria and their descendants colonizing the human gut may disrupt the balance of the local microbiota and become pathogenic. However, the safety zone for the abundance of these taxa is not predictable. Therefore, Deltaproteobacteria and their descendants may be protective or harmful. The necessary equilibrium of these taxa in the human gut requires further investigation.

Organisms that are causally related to PBC (12, 14, 74), such as *Streptococcus*, *Enterococcus*, or *Veillonella*, were not identified as potential biomarkers in this study. The alterations in the GM associated with PBC may be an effect rather than a cause, indicating that the disease state of PBC (bile stasis and immune dysregulation of the gut-liver axis) affects the composition of the GM.

The efficacies of treatment options for PBC are limited (1, 75). Microbiome modulation therapies, such as the use of antibiotics, supplementation with probiotics, and fecal microbiota transplantation (FMT), have been used to treat liver diseases, including recurrent encephalopathy, non-alcoholic fatty liver disease, liver cancer, and cholestatic liver diseases (76–79). Although no clinical trials have specifically focused on PBC, promising results have been obtained regarding the safety of FMT in primary sclerosing cholangitis (PSC), another cholestatic liver disease. A previous study demonstrated improvements in the alkaline phosphatase levels and enrichment of specific bacterial strains in patients with PSC (80). Therefore, the identification of bacteria that could be protective or potentially harmful against PBC may be a therapeutic intervention for the regulation of the GM.

This study has advantages and limitations. It is the first bi-directional, two-sample MR study to explore the causal relationship between the GM and PBC, with strict conditions for screening the IVs. This study provides genetic evidence regarding the gut-liver axis and identifies three bacterial taxa associated with PBC that have not been previously studied. However, the number of microbiota taxa in the database was limited, resulting in a lack of IVs for the MR analysis. In addition, it is unclear whether there are overlapping samples in the GWAS data of GM and PBC, which may lead to bias. Further experimental and clinical validation is necessary to confirm these findings. Last, the GWAS samples of the MiBioGen database were mainly of European ancestry; therefore, these results are limited to patients of European descent.

In conclusion, this study identified a causal relationships between the GM and PBC. The class Coriobacteriia and order Coriobacteriales may be intermediate factors in inducing PBC and participating in disease progression, whereas Deltaproteobacteria may play a protective role. However, it is essential to note that the findings of this study have limitations, and further validation through experiments and clinical studies is required to confirm these observations.

## Data availability statement

The original contributions presented in the study are included in the article/**Supplementary Material**. Further inquiries can be directed to the corresponding author.

## Ethics statement

Each cohort obtained ethical approval and consent to participate in accordance with their local regulations and institute requirements (mentioned in previous studies). The current study remained within the limits of the ethical committee’s original permission. The studies were conducted in accordance with the local legislation and institutional requirements. The human samples used in this study were acquired from gifted from another research group. Written informed consent for participation was not required from the participants or the participants’ legal guardians/next of kin in accordance with the national legislation and institutional requirements.

## Author contributions

XL and XY contributed to the study design. XL performed data collecting, computations, and manuscript writing. XY and XL contributed to the article and approved the submitted version.

## Appendices

(Eq.A)
$${\text{R}}^{2}=\frac{2\times \text{EAF}\times \left(1-\text{EAF}\right)\times {\text{β}}_{\text{exposure}}^{2}}{2\times \text{EAF}\times \left(1-\text{EAF}\right)\times {\text{β}}_{\text{exposure}}^{2}+2\times \text{EAF}\times \left(1-\text{EAF}\right)\times \text{N}\times {\text{SE}}_{\text{exposure}}^{2}}\;$$

(Eq.B)
$$\text{F}={\text{R}}^{2}\times \frac{\text{N}-2}{1-{\text{R}}^{2}}\;$$

${\text{R}}^{2}$ stands for the exposure variance defined by each IV, EAF means effect allele frequency, ${\text{β}}_{\text{exposure}}$ and ${\text{SE}}_{\text{exposure}}^{2}$ refer to the estimated effect and standard error of SNPs on specific gut microbiome respectively, and N is the sample size.

## References

[1] Lindor KD Bowlus CL Boyer J Levy C Mayo M. Primary biliary cholangitis: 2018 practice guidance from the american association for the study of liver diseases. Hepatology (2019) 69(1):394–419. doi: 10.1002/hep.30145 30070375

[2] Pabst O Hornef MW Schaap FG Cerovic V Clavel T Bruns T. Gut-liver axis: barriers and functional circuits. Nat Rev Gastroenterol Hepatol (2023) 20(7):447–61. doi: 10.1038/s41575-023-00771-6 37085614

[3] Di Ciaula A Baj J Garruti G Celano G De Angelis M Wang HH. Liver steatosis, gut-liver axis, microbiome and environmental factors. A never-ending bidirectional cross-talk. J Clin Med (2020) 9(8):2648. doi: 10.3390/jcm9082648 32823983 PMC7465294

[4] Gudan A Jamioł-Milc D Hawryłkowicz V Skonieczna-Żydecka K Stachowska E. The prevalence of small intestinal bacterial overgrowth in patients with non-alcoholic liver diseases: nafld, nash, fibrosis, cirrhosis-a systematic review, meta-analysis and meta-regression. Nutrients (2022) 14(24):5261. doi: 10.3390/nu14245261 36558421 PMC9783356

[5] Thaiss CA Zmora N Levy M Elinav E. The microbiome and innate immunity. Nature (2016) 535(7610):65–74. doi: 10.1038/nature18847 27383981

[6] Carpino G Del Ben M Pastori D Carnevale R Baratta F Overi D. Increased liver localization of lipopolysaccharides in human and experimental nafld. Hepatology (2020) 72(2):470–85. doi: 10.1002/hep.31056 31808577

[7] Chen J Thomsen M Vitetta L. Interaction of gut microbiota with dysregulation of bile acids in the pathogenesis of nonalcoholic fatty liver disease and potential therapeutic implications of probiotics. J Cell Biochem (2019) 120(3):2713–20. doi: 10.1002/jcb.27635 30443932

[8] Allen K Jaeschke H Copple BL. Bile acids induce inflammatory genes in hepatocytes: A novel mechanism of inflammation during obstructive cholestasis. Am J Pathol (2011) 178(1):175–86. doi: 10.1016/j.ajpath.2010.11.026 PMC3070591 21224055

[9] Manley S Ni HM Kong B Apte U Guo G Ding WX. Suppression of autophagic flux by bile acids in hepatocytes. Toxicol Sci (2014) 137(2):478–90. doi: 10.1093/toxsci/kft246 PMC3908720 24189133

[10] Zhu L Baker SS Gill C Liu W Alkhouri R Baker RD. Characterization of gut microbiomes in nonalcoholic steatohepatitis (Nash) patients: A connection between endogenous alcohol and nash. Hepatology (2013) 57(2):601–9. doi: 10.1002/hep.26093 23055155

[11] Chen J Vitetta L. The role of butyrate in attenuating pathobiont-induced hyperinflammation. Immune Netw (2020) 20(2):e15. doi: 10.4110/in.2020.20.e15 32395367 PMC7192831

[12] Tang R Wei Y Li Y Chen W Chen H Wang Q. Gut microbial profile is altered in primary biliary cholangitis and partially restored after udca therapy. Gut (2018) 67(3):534–41. doi: 10.1136/gutjnl-2016-313332 28213609

[13] Liwinski T Casar C Ruehlemann MC Bang C Sebode M Hohenester S. A disease-specific decline of the relative abundance of bifidobacterium in patients with autoimmune hepatitis. Aliment Pharmacol Ther (2020) 51(12):1417–28. doi: 10.1111/apt.15754 32383181

[14] Lv LX Fang DQ Shi D Chen DY Yan R Zhu YX. Alterations and correlations of the gut microbiome, metabolism and immunity in patients with primary biliary cirrhosis. Environ Microbiol (2016) 18(7):2272–86. doi: 10.1111/1462-2920.13401 27243236

[15] Furukawa M Moriya K Nakayama J Inoue T Momoda R Kawaratani H. Gut dysbiosis associated with clinical prognosis of patients with primary biliary cholangitis. Hepatol Res (2020) 50(7):840–52. doi: 10.1111/hepr.13509 32346970

[16] Rinninella E Raoul P Cintoni M Franceschi F Miggiano GAD Gasbarrini A. What is the healthy gut microbiota composition? A changing ecosystem across age, environment, diet, and diseases. Microorganisms (2019) 7(1):14. doi: 10.3390/microorganisms7010014 30634578 PMC6351938

[17] Terziroli Beretta-Piccoli B Mieli-Vergani G Vergani D Vierling JM Adams D Alpini G. The challenges of primary biliary cholangitis: what is new and what needs to be done. J Autoimmun (2019) 105:102328. doi: 10.1016/j.jaut.2019.102328 31548157

[18] Greenland S. An introduction to instrumental variables for epidemiologists. Int J Epidemiol (2000) 29(4):722–9. doi: 10.1093/ije/29.4.722 10922351

[19] Burgess S Thompson SG. Mendelian Randomization: Methods for Causal Inference Using Genetic Variants. New York: CRC Press (2021).

[20] Bowden J Davey Smith G Burgess S. Mendelian randomization with invalid instruments: effect estimation and bias detection through egger regression. Int J Epidemiol (2015) 44(2):512–25. doi: 10.1093/ije/dyv080 PMC4469799 26050253

[21] Wang J Kurilshikov A Radjabzadeh D Turpin W Croitoru K Bonder MJ. Meta-analysis of human genome-microbiome association studies: the mibiogen consortium initiative. Microbiome (2018) 6(1):101. doi: 10.1186/s40168-018-0479-3 29880062 PMC5992867

[22] Kurilshikov A Medina-Gomez C Bacigalupe R Radjabzadeh D Wang J Demirkan A. Large-scale association analyses identify host factors influencing human gut microbiome composition. Nat Genet (2021) 53(2):156–65. doi: 10.1038/s41588-020-00763-1 PMC8515199 33462485

[23] Kurilshikov A. Mibiogen Miqtl Pipeline: Github (2018). Available at: https://github.com/alexa-kur/miQTL_cookbook.

[24] Cordell HJ Fryett JJ Ueno K Darlay R Aiba Y Hitomi Y. An international genome-wide meta-analysis of primary biliary cholangitis: novel risk loci and candidate drugs. J Hepatol (2021) 75(3):572–81. doi: 10.1016/j.jhep.2021.04.055 PMC8811537 34033851

[25] Sanna S van Zuydam NR Mahajan A Kurilshikov A Vich Vila A Võsa U. Causal relationships among the gut microbiome, short-chain fatty acids and metabolic diseases. Nat Genet (2019) 51(4):600–5. doi: 10.1038/s41588-019-0350-x PMC6441384 30778224

[26] Cao J Wang N Luo Y Ma C Chen Z Chenzhao C. A cause–effect relationship between graves’ Disease and the gut microbiome contributes to the thyroid–gut axis: A bidirectional two-sample mendelian randomization study. Front Immunol (2023) 14:977587. doi: 10.3389/fimmu.2023.977587 36865531 PMC9974146

[27] Li P Wang H Guo L Gou X Chen G Lin D. Association between gut microbiota and preeclampsia-eclampsia: A two-sample mendelian randomization study. BMC Med (2022) 20(1):443. doi: 10.1186/s12916-022-02657-x 36380372 PMC9667679

[28] Kamat MA Blackshaw JA Young R Surendran P Burgess S Danesh J. Phenoscanner V2: an expanded tool for searching human genotype–phenotype associations. Bioinformatics (2019) 35(22):4851–3. doi: 10.1093/bioinformatics/btz469 PMC6853652 31233103

[29] Staley JR Blackshaw J Kamat MA Ellis S Surendran P Sun BB. Phenoscanner: A database of human genotype–phenotype associations. Bioinformatics (2016) 32(20):3207–9. doi: 10.1093/bioinformatics/btw373 PMC5048068 27318201

[30] Lawlor DA Harbord RM Sterne JA Timpson N Davey Smith G. Mendelian randomization: using genes as instruments for making causal inferences in epidemiology. Stat Med (2008) 27(8):1133–63. doi: 10.1002/sim.3034 17886233

[31] Burgess S Thompson SG. Avoiding bias from weak instruments in mendelian randomization studies. Int J Epidemiol (2011) 40(3):755–64. doi: 10.1093/ije/dyr036 21414999

[32] Shim H Chasman DI Smith JD Mora S Ridker PM Nickerson DA. A multivariate genome-wide association analysis of 10 ldl subfractions, and their response to statin treatment, in 1868 caucasians. PloS One (2015) 10(4):e0120758. doi: 10.1371/journal.pone.0120758 25898129 PMC4405269

[33] Burgess S Butterworth A Thompson SG. Mendelian randomization analysis with multiple genetic variants using summarized data. Genet Epidemiol (2013) 37(7):658–65. doi: 10.1002/gepi.21758 PMC4377079 24114802

[34] Bowden J Davey Smith G Haycock PC Burgess S. Consistent estimation in mendelian randomization with some invalid instruments using a weighted median estimator. Genet Epidemiol (2016) 40(4):304–14. doi: 10.1002/gepi.21965 PMC4849733 27061298

[35] Hemani G Zheng J Elsworth B Wade KH Haberland V Baird D. The mr-base platform supports systematic causal inference across the human phenome. Elife (2018) 7:e34408. doi: 10.7554/eLife.34408 29846171 PMC5976434

[36] Hartwig FP Davey Smith G Bowden J. Robust inference in summary data mendelian randomization via the zero modal pleiotropy assumption. Int J Epidemiol (2017) 46(6):1985–98. doi: 10.1093/ije/dyx102 PMC5837715 29040600

[37] Slob EAW Burgess S. A comparison of robust mendelian randomization methods using summary data. Genet Epidemiol (2020) 44(4):313–29. doi: 10.1002/gepi.22295 PMC7317850 32249995

[38] Long Y Tang L Zhou Y Zhao S Zhu H. Causal relationship between gut microbiota and cancers: A two-sample mendelian randomisation study. BMC Med (2023) 21(1):66. doi: 10.1186/s12916-023-02761-6 36810112 PMC9945666

[39] Hemani G Tilling K Davey Smith G. Orienting the causal relationship between imprecisely measured traits using gwas summary data. PloS Genet (2017) 13(11):e1007081. doi: 10.1371/journal.pgen.1007081 29149188 PMC5711033

[40] Greco MF Minelli C Sheehan NA Thompson JR. Detecting pleiotropy in mendelian randomisation studies with summary data and a continuous outcome. Stat Med (2015) 34(21):2926–40. doi: 10.1002/sim.6522 25950993

[41] Bowden J Del Greco MF Minelli C Zhao Q Lawlor DA Sheehan NA. Improving the accuracy of two-sample summary-data mendelian randomization: moving beyond the nome assumption. Int J Epidemiol (2019) 48(3):728–42. doi: 10.1093/ije/dyy258 PMC6659376 30561657

[42] Team RC. R: A Language and Environment for Statistical Computing: R Foundation for Statistical Computing (2018). Available at: https://www.R-project.org/.

[43] Verbanck M Chen C-Y Neale B Do R. Detection of widespread horizontal pleiotropy in causal relationships inferred from mendelian randomization between complex traits and diseases. Nat Genet (2018) 50(5):693–8. doi: 10.1038/s41588-018-0099-7 PMC6083837 29686387

[44] Wang R Tang R Li B Ma X Schnabl B Tilg H. Gut microbiome, liver immunology, and liver diseases. Cell Mol Immunol (2021) 18(1):4–17. doi: 10.1038/s41423-020-00592-6 33318628 PMC7852541

[45] Li Y Tang R Leung PSC Gershwin ME Ma X. Bile acids and intestinal microbiota in autoimmune cholestatic liver diseases. Autoimmun Rev (2017) 16(9):885–96. doi: 10.1016/j.autrev.2017.07.002 28698093

[46] Rojas M Restrepo-Jiménez P Monsalve DM Pacheco Y Acosta-Ampudia Y Ramírez-Santana C. Molecular mimicry and autoimmunity. J Autoimmun (2018) 95:100–23. doi: 10.1016/j.jaut.2018.10.012 30509385

[47] Bogdanos DP Baum H Grasso A Okamoto M Butler P Ma Y. Microbial mimics are major targets of crossreactivity with human pyruvate dehydrogenase in primary biliary cirrhosis. J Hepatol (2004) 40(1):31–9. doi: 10.1016/s0168-8278(03)00501-4 14672611

[48] Selmi C Balkwill DL Invernizzi P Ansari AA Coppel RL Podda M. Patients with primary biliary cirrhosis react against a ubiquitous xenobiotic-metabolizing bacterium. Hepatology (2003) 38(5):1250–7. doi: 10.1053/jhep.2003.50446 14578864

[49] Mattner J Savage PB Leung P Oertelt SS Wang V Trivedi O. Liver autoimmunity triggered by microbial activation of natural killer T cells. Cell Host Microbe (2008) 3(5):304–15. doi: 10.1016/j.chom.2008.03.009 PMC2453520 18474357

[50] Wang JJ Yang GX Zhang WC Lu L Tsuneyama K Kronenberg M. Escherichia coli infection induces autoimmune cholangitis and anti-mitochondrial antibodies in non-obese diabetic (Nod).B6 (Idd10/idd18) mice. Clin Exp Immunol (2014) 175(2):192–201. doi: 10.1111/cei.12224 24128311 PMC3892410

[51] Ludwig W Euzéby J Schumann P Busse H-J Trujillo ME Kämpfer P. Road map of the phylum actinobacteria. In: Bergey’s Manual^®^ of Systematic Bacteriology. New York: Springer New York, NY (2012). p. 1–28.

[52] Würdemann D Tindall BJ Pukall R Lünsdorf H Strömpl C Namuth T. Gordonibacter pamelaeae gen. Nov., sp. Nov., a new member of the coriobacteriaceae isolated from a patient with crohn’s disease, and reclassification of eggerthella hongkongensis lau et al. 2006 as paraeggerthella hongkongensis gen. Nov., comb. Nov. Int J Syst Evol Microbiol (2009) 59(Pt 6):1405–15. doi: 10.1099/ijs.0.005900-0 19502325

[53] Stackebrandt E Zeytun A Lapidus A Nolan M Lucas S Hammon N. Complete genome sequence of coriobacterium glomerans type strain (Pw2(T)) from the midgut of pyrrhocoris apterus L. (Red soldier bug). Stand Genomic Sci (2013) 8(1):15–25. doi: 10.4056/sigs.3507020 23961308 PMC3739169

[54] Mavrommatis K Pukall R Rohde C Chen F Sims D Brettin T. Complete genome sequence of cryptobacterium curtum type strain (12-3). Stand Genomic Sci (2009) 1(2):93–100. doi: 10.4056/sigs.12260 21304644 PMC3035227

[55] Gupta RS Chen WJ Adeolu M Chai Y. Molecular signatures for the class coriobacteriia and its different clades; proposal for division of the class coriobacteriia into the emended order coriobacteriales, containing the emended family coriobacteriaceae and atopobiaceae fam. Nov., and eggerthellales ord. Nov., containing the family eggerthellaceae fam. Nov. Int J Syst Evol Microbiol (2013) 63(Pt 9):3379–97. doi: 10.1099/ijs.0.048371-0 23524353

[56] Yi W Ji Y Gao H Pan R Wei Q Cheng J. Does the gut microbiome partially mediate the impact of air pollutants exposure on liver function? Evidence based on schizophrenia patients. Environ pollut (2021) 291:118135. doi: 10.1016/j.envpol.2021.118135 34534831

[57] Wu Y Tan MB Jiang HY Lu XD Li XW Yang FS. [Microorganisms in the urethral secretions of chronic prostatitis patients: A genomic analysis]. Zhonghua Nan Ke Xue (2021) 27(2):114–23. 34914326

[58] Sorokin A Brown JL Thompson PD. Primary biliary cirrhosis, hyperlipidemia, and atherosclerotic risk: A systematic review. Atherosclerosis (2007) 194(2):293–9. doi: 10.1016/j.atherosclerosis.2006.11.036 17240380

[59] Carey EJ Ali AH Lindor KD. Primary biliary cirrhosis. Lancet (2015) 386(10003):1565–75. doi: 10.1016/s0140-6736(15)00154-3 26364546

[60] Guo L Wang YY Wang JH Zhao HP Yu Y Wang GD. Associations of gut microbiota with dyslipidemia based on sex differences in subjects from northwestern China. World J Gastroenterol (2022) 28(27):3455–75. doi: 10.3748/wjg.v28.i27.3455 PMC9346449 36158270

[61] Storm-Larsen C Hande LN Kummen M Thunhaug H Vestad B Hansen SH. Reduced gut microbial diversity in familial hypercholesterolemia with no effect of omega-3 polyunsaturated fatty acids intervention - a pilot trial. Scand J Clin Lab Invest (2022) 82(5):363–70. doi: 10.1080/00365513.2022.2102540 35913798

[62] Floreani A Cazzagon N Franceschet I Canesso F Salmaso L Baldo V. Metabolic syndrome associated with primary biliary cirrhosis. J Clin Gastroenterol (2015) 49(1):57–60. doi: 10.1097/mcg.0000000000000029 24231935

[63] Waite DW ChuvoChina M Pelikan C Parks DH Yilmaz P Wagner M. Proposal to reclassify the proteobacterial classes deltaproteobacteria and oligoflexia, and the phylum thermodesulfobacteria into four phyla reflecting major functional capabilities. Int J Syst Evol Microbiol (2020) 70(11):5972–6016. doi: 10.1099/ijsem.0.004213 33151140

[64] Campbell AG Campbell JH Schwientek P Woyke T Sczyrba A Allman S. Multiple single-cell genomes provide insight into functions of uncultured deltaproteobacteria in the human oral cavity. PloS One (2013) 8(3):e59361. doi: 10.1371/journal.pone.0059361 23555659 PMC3608642

[65] Amado PPP Kawamoto D Albuquerque-Souza E Franco DC Saraiva L Casarin RCV. Oral and fecal microbiome in molar-incisor pattern periodontitis. Front Cell Infect Microbiol (2020) 10:583761. doi: 10.3389/fcimb.2020.583761 33117737 PMC7578221

[66] Wei B Wang Y Xiang S Jiang Y Chen R Hu N. Alterations of gut microbiome in patients with type 2 diabetes mellitus who had undergone cholecystectomy. Am J Physiol Endocrinol Metab (2021) 320(1):E113–e21. doi: 10.1152/ajpendo.00471.2020 33166187

[67] Natividad JM Lamas B Pham HP Michel ML Rainteau D Bridonneau C. Bilophila wadsworthia aggravates high fat diet induced metabolic dysfunctions in mice. Nat Commun (2018) 9(1):2802. doi: 10.1038/s41467-018-05249-7 30022049 PMC6052103

[68] Coutinho C Coutinho-Silva R Zinkevich V Pearce CB Ojcius DM Beech I. Sulphate-reducing bacteria from ulcerative colitis patients induce apoptosis of gastrointestinal epithelial cells. Microb Pathog (2017) 112:126–34. doi: 10.1016/j.micpath.2017.09.054 28963010

[69] Lin YC Lin HF Wu CC Chen CL Ni YH. Pathogenic effects of desulfovibrio in the gut on fatty liver in diet-induced obese mice and children with obesity. J Gastroenterol (2022) 57(11):913–25. doi: 10.1007/s00535-022-01909-0 35976494

[70] Murros KE. Hydrogen sulfide produced by gut bacteria may induce parkinson’s disease. Cells (2022) 11(6):978. doi: 10.3390/cells11060978 35326429 PMC8946538

[71] Karnachuk OV Rusanov II Panova IA Grigoriev MA Zyusman VS Latygolets EA. Microbial sulfate reduction by desulfovibrio is an important source of hydrogen sulfide from a large swine finishing facility. Sci Rep (2021) 11(1):10720. doi: 10.1038/s41598-021-90256-w 34021225 PMC8140134

[72] Buret AG Allain T Motta JP Wallace JL. Effects of hydrogen sulfide on the microbiome: from toxicity to therapy. Antioxid Redox Signal (2022) 36(4-6):211–9. doi: 10.1089/ars.2021.0004 PMC8861923 33691464

[73] Chen L Gao Y Zhao Y Yang G Wang C Zhao Z. Chondroitin sulfate stimulates the secretion of H(2)S by desulfovibrio to improve insulin sensitivity in nafld mice. Int J Biol Macromol (2022) 213:631–8. doi: 10.1016/j.ijbiomac.2022.05.195 35667460

[74] Chen W Wei Y Xiong A Li Y Guan H Wang Q. Comprehensive analysis of serum and fecal bile acid profiles and interaction with gut microbiota in primary biliary cholangitis. Clin Rev Allergy Immunol (2020) 58(1):25–38. doi: 10.1007/s12016-019-08731-2 30900136

[75] Lindor KD Bowlus CL Boyer J Levy C Mayo M. Primary biliary cholangitis: 2021 practice guidance update from the american association for the study of liver diseases. Hepatology (2022) 75(4):1012–3. doi: 10.1002/hep.32117 34431119

[76] Del Barrio M Lavín L Santos-Laso Á Arias-Loste MT Odriozola A Rodriguez-Duque JC. Faecal microbiota transplantation, paving the way to treat non-alcoholic fatty liver disease. Int J Mol Sci (2023) 24(7):6123. doi: 10.3390/ijms24076123 37047094 PMC10094628

[77] Suk KT Koh H. New perspective on fecal microbiota transplantation in liver diseases. J Gastroenterol Hepatol (2022) 37(1):24–33. doi: 10.1111/jgh.15729 34734433

[78] Yan X Bai L Qi P Lv J Song X Zhang L. Potential effects of regulating intestinal flora on immunotherapy for liver cancer. Int J Mol Sci (2023) 24(14):11387. doi: 10.3390/ijms241411387 37511148 PMC10380345

[79] Gerussi A D’Amato D Cristoferi L O’Donnell SE Carbone M Invernizzi P. Multiple therapeutic targets in rare cholestatic liver diseases: time to redefine treatment strategies. Ann Hepatol (2020) 19(1):5–16. doi: 10.1016/j.aohep.2019.09.009 31771820

[80] Allegretti JR Kassam Z Carrellas M Mullish BH Marchesi JR Pechlivanis A. Fecal microbiota transplantation in patients with primary sclerosing cholangitis: A pilot clinical trial. Off J Am Coll Gastroenterol | ACG (2019) 114(7):1071–9. doi: 10.14309/ajg.0000000000000115 30730351

